# The association between whole-blood lead concentration and all-cause or cardiovascular disease mortality in hypertensive patients

**DOI:** 10.1038/s41598-025-22009-y

**Published:** 2025-11-03

**Authors:** Yan Pu, Qiang Zheng, Ting Yi, Xianming Tang

**Affiliations:** 1 Department of Emergency Medicine, The Second Xiangya Hospital, Central South University, Changsha, 410011 Hunan China; 2 Emergency and Difficult Diseases, Institute of Central South University, Changsha, 410011 Hunan China; 3 Emergency Department, Sichuan Academy of Medical Sciences & Sichuan Provincial People’s Hospital, Chengdu, 610000 Sichuan China; 4 Department of Cardiovascular Medicine, The Second Xiangya Hospital, Central South University, Changsha, 410011 Hunan China

**Keywords:** Lead, Hypertension, Subgroups, All-cause mortality, Cardiovascular mortality, NHANES, Biochemistry, Environmental sciences, Health care

## Abstract

The purpose of this study was to examine the relationship between whole-blood lead levels and mortality in a hypertensive population in the United States. In our study, 8364 subjects aged 20 years and older were included. We used a weighted COX proportional risk regression model to calculate risk ratios (HR) and 95% confidence intervals (CI) for blood lead and mortality. The relationship between whole-blood lead levels and mortality was described by a restricted cubic spline curve. Kaplan Meier curves were used to describe the relationship between survival time and survival in study subjects, and all-cause mortality was analyzed in subgroups. Using the lowest quartile (Q1) as a reference, the HRs for all-cause mortality in model 3 (Q2, Q3, and Q4) were 1.05 (95% CI 0.84–1.32), 1.10 (95% CI 0.89–1.36), and 1.44 (95% CI 1.16–1.79), respectively. For cardiovascular mortality, they were 1.03 (95% CI 0.62–1.52), 1.30 (95% CI 0.77–2.21), and 1.97 (95% CI 1.31–2.97), respectively. Weighted restricted cubic spline regression confirmed a positive correlation between whole-blood lead levels and risk ratio (HR) (P-overall < 0.001). Weighted Kaplan–Meier curves showed a significant downward trend in survival in the hypertensive population with increasing whole-blood lead levels. (*P* < 0.0001 for all log-rankings). Subgroup analysis of all-cause mortality showed a strong positive association between whole-blood lead levels and all-cause mortality in different populations. Whole-blood lead concentration showed a non-linear positive correlation with all-cause mortality and cardiovascular mortality in hypertensive patients.

**Supplementary Information:**

The online version contains supplementary material available at 10.1038/s41598-025-22009-y.

## Introduction

Lead is a heavy metal that can cause serious damage to the nervous, digestive, and blood systems of the body. Hypertension is one of the most prominent risk factors for the occurrence of myocardial infarction, stroke and kidney failure, and even death. Lead poisoning causes heart and blood vessel damage and increases the risk of cardiovascular disease^1^. Potential mechanisms include enhanced lead-induced oxidative stress, disturbed lipid metabolism, and decreased production of nitric oxide and guanylate cyclase^2,3^. The relationship between hypertension and whole-blood lead is unclear. Several studies have shown that elevated whole-blood lead levels significantly increase the prevalence of hypertension^4–6^, and a recent large cross-sectional study of a US population showed a nonsignificant correlation between blood lead concentrations and hypertension^7^. Another large study of a Gulf Coast follow-up population showed no significant association between lead concentration in the body and hypertension^8^. However, many studies have shown that higher lead concentrations are associated with increased mortality. A large classical cohort study based on the U.S. population showed that increased blood lead levels significantly increased all-cause mortality and cardiovascular disease mortality in the study population^9^. A study based on the NHANES database showed that blood lead levels were positively associated with the risk of total mortality and cardiovascular disease mortality in the population^10–12^. Studies in diabetic populations have shown that elevated blood lead concentrations increase the risk of death in diabetic patients^13^. The association between blood lead levels and all-cause mortality and cardiovascular disease mortality in the US hypertensive population has not been studied, so we designed this study. Although several NHANES-based studies have linked blood lead to mortality in the general United States population^14–16^, evidence specifically addressing hypertensive adults remains scarce and inconsistent. Only four prior analyses restricted to hypertensive participants were identified; all were limited by short follow-up (≤ 10 years), failure to model non-linear dose-response relationships, or lack of adjustment for key confounders such as antihypertensive intensity and co-exposure to cadmium. Importantly, hypertension amplifies lead-induced endothelial dysfunction and oxidative stress through additive activation of the renin-angiotensin system and increased arterial stiffness, suggesting that the dose-response curve and absolute risk may differ from the general population. We therefore conducted this focused investigation to quantify the shape and magnitude of the blood lead–mortality association among hypertensive adults, aiming to inform targeted public-health strategies.Based on data from the NHANES database of hypertensive adults from 2009 to 2016, the relationship between whole-blood lead levels and mortality was analyzed using a weighted COX proportional risk regression model, and a subgroup analysis of all-cause mortality was performed to assess the robustness of this association.

## Materials and methods

### Study population

**Fig. 1:**
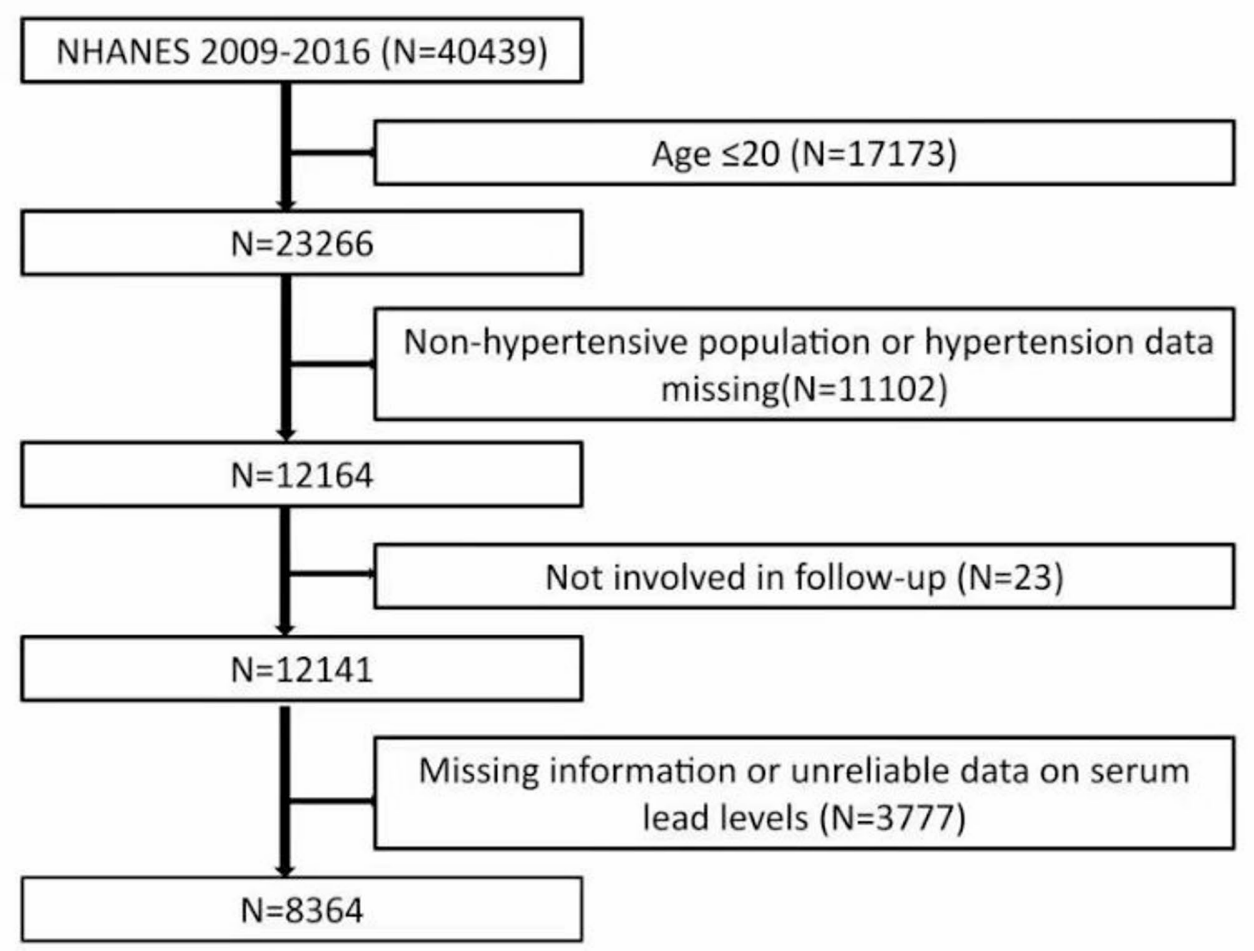
Flowchart.

We obtained data from the NHANES database for the 2009–2016 survey cycle for this retrospective cohort study with a total of 40,439 participants, and we included participants younger than or equal to 20 years of age (*n* = 17,173), non-hypertensives, and participants with missing information on hypertension (*n* = 11,102), participants with missing information on follow-up (*n* = 23), participants with serum participants with missing information or unreliable data on lead levels (*n* = 3777) were excluded. As a result, a total of 8364 participants were included in the final analysis, and Fig. 1 illustrates the specific inclusion and exclusion process. Approval of the study protocol was granted by the Institutional Review Board of the US Centers for Disease Control. Written informed consent was provided by each study subject.

### Determination of whole-blood lead

Lead concentration in blood was selected as the subject of our study and was determined in serum by inductively coupled plasma-dynamic reaction cell-mass spectrometry (ICP-DRC-MS) after a standard blood sampling, and storage procedure. Detailed instructions for sample collection and handling are available in the NHANES Laboratory/Medical Technology Procedures Manual (LPM)^17^. Measurements below the limit of detection (LOD) for blood lead were replaced by LOD divided by the square root of 2^18^. All blood lead concentrations are reported in µg/dL, as measured by NHANES using inductively coupled plasma mass spectrometry (ICP-DRC-MS). The values are not converted to µmol/L, and the elevated levels are consistent with the study population’s exposure profile.

### Definition of covariates

A standard questionnaire was used to collect demographic information on the study population. Other confounders included: smokers were those who had consumed at least 100 cigarettes over their lifetime^19,20^. People with fasting glucose levels higher than 126 mg/dL or 2-h blood glucose levels above 200 mg/dL (measured by oral glucose tolerance test) or treated with insulin or antidiabetic drugs were diabetic^21^. Participants with hypertension are those who have an average systolic blood pressure of more than or equal to 130 mmHg and average diastolic blood pressure of more than or equal to 80 mmHg, or who have been medically diagnosed with hypertension or are taking hypertensive drugs to reduce their blood pressure^12^. BMI is measured by taking the weight in kilograms and dividing it by the square of the height in square meters^22^. Drinkers were defined as participants who consumed alcohol more than or equal to 12 times per year. Standard biochemical techniques were used to assess total cholesterol, high-density lipoprotein cholesterol (HDL) and glycated hemoglobin levels in the NHANES database. The laboratory methods documentation section of NHANES contains a comprehensive overview of the laboratory procedures performed. Random forest interpolation was performed for covariates with missing values^23^.

### Definition of death

Mortality data for study subjects were downloaded through the NHANES database’s public use link mortality file^24^. All-cause mortality was determined as a death from any cause, and when codes I00-I09, I11, I13, I20-I51, or I60-I69 were used, study subjects were defined as cardiovascular disease deaths^25^.

### Statistical analysis

The NHANES database uses a complex sampling design method, so we used appropriate weights in the statistical analysis. In the baseline information, the mean ± standard deviation was used to represent continuous variables and percentages were used for the categorical variables. Weighted ANOVA and weighted chi-square tests were used for comparing between-group differences in continuous and categorical variables, respectively. Furthermore, the study subjects were classified into four groups based on the quartiles of their serum lead concentrations, The lowest quartile group Q1 was used as the reference group. To adjust for the effect of confounding factors, we calculated risk ratios (HR) and 95% confidence intervals (CI) for blood lead and mortality using a weighted COX proportional risk regression model. Model adjustments were performed, Model 1: no adjustment. Model 2: adjusted for demographic information (age, gender, and race), and Model 3: adjusted for information on all covariates (age, gender, race, marriage, education level, BMI, HDL, triglycerides, glycohemoglobin, smoking status and diabetes.). A weighted restricted cubic spline curve was used to visualize the relationship between whole-blood lead concentrations and total and cardiovascular mortality. We used weighted Kaplan Meier curves to describe the relationship between survival time and survival of the study subjects. A subgroup analysis of all-cause mortality was also performed with the purpose of discussing the stability of the connection between whole-blood lead concentrations and all-cause mortality in different populations and discussing the interaction between different covariates by likelihood ratio tests. We used R4.21 version for all analyses^13,26–28^.

## Results

### Baseline information

The study ultimately included 8364 patients with hypertension who met the criteria, of whom 48.3% were male and 51.7% were female, with a mean BMI of 30.66 ± 7.15 kg/m^2^, 48.1% were smokers, 22.3% were diabetics, and 56.5% were alcohol drinkers. The study population was divided into four groups based on whole-blood lead levels Q1 (< 0.85 µg/dL), Q2 (0.85 to 1.28 µg/dL), Q3 (1.28 to 2.00 µg/dL), and Q4 (≥ 2.00 µg/dL). For the indicators we focused on, all-cause mortality and cardiovascular mortality in the total population were 11.9% and 3.6%, respectively, with increasing whole-blood lead levels, all-cause mortality (Q1: 5.2%, Q2: 10.3%, Q3: 13.2%, Q4. 21.3%) and cardiovascular mortality (Q1: 1.4%, Q2: 2.5%, Q3: 4.1%, Q4. 7.3%) showed a continuous upward trend. The baseline information is shown in Table 1.

**Table 1:** Baseline information, weighted. Mean ± standard error (SE) for continuous variables, percentages (%) for categorical variables. BMI, body mass index; HDL, high-density lipoprotein; TC, total cholesterol; GFR, glomerular rate filtration; CVD death, cardiovascular disease death.

Characteristic	Whole-blood lead				
	Overall	Q1	Q2	Q3	Q4
Blood lead range (µg/dL)		0.050–0.850	0.850–1.280	1.280-2.000	2.000-6.680
N	8364	2070	2105	2095	2094
Age, n (%)					
20–40 years	20.5	40.3	19.3	10.3	7.6
40-60years	41.0	39.8	42.3	42.5	39.5
> 60years	38.4	19.9	38.4	47.2	52.9
Gender, n(%)					
Male	48.3	56.0	51.2	45.6	37.8
Female	51.7	44.0	48.8	54.4	62.2
Race, n(%)					
Mexican America	6.5	8.0	6.2	6.1	5.4
Non-Hispanic Black	12.9	12.9	11.7	12.8	14.2
Non-Hispanic White	69.1	67.1	69.5	70.2	69.9
Other races	11.7	12.5	12.6	11.0	10.5
Marriage, n(%)					
Married/living with partner	63.3	63.0	61.4	66.2	62.6
Widowed/divorced/separated	23.9	18.1	25.7	24.4	28.9
Never married	12.8	18.8	12.9	9.4	8.5
Education, n (%)					
< High school	18.3	13.3	16.9	19.8	24.5
High school	23.3	22.8	23.6	22.0	24.6
> High school	58.5	63.8	59.5	57.9	50.9
BMI, kg/m^2^	30.66 ± 7.15	32.99 ± 8.09	30.91 ± 6.99	29.77 ± 6.32	28.32 ± 5.86
Hdl, mg/dl	52.45 ± 17.24	49.23 ± 14.61	51.36 ± 15.57	54.54 ± 19.25	55.63 ± 18.99
TC, mg/dl	197.78 ± 41.94	191.84 ± 39.42	199.44 ± 43.80	200.86 ± 42.36	200.13 ± 41.89
Triglyceride, mg/dl	144.84 ± 91.02	149.86 ± 92.68	150.31 ± 104.62	134.82 ± 80.55	139.18 ± 78.58
GFR, ml/min/1.73 m^2^	86.59 ± 25.19	94.65 ± 27.36	85.86 ± 23.05	83.63 ± 22.92	80.22 ± 24.41
Smoking status, n(%)					
NO	51.9	63.6	55.1	48.5	36.5
YES	48.1	36.4	44.9	51.5	63.5
Diabetes, n(%)					
NO	77.7	77.0	74.6	80.3	79.4
YES	22.3	23.0	25.4	19.7	20.6
Alcohol status, n(%)					
No	43.5	50.8	44.1	40.4	35.8
Yes	56.5	49.2	55.9	59.6	64.2
All deaths, n(%)	11.9	5.2	10.3	13.2	21.3
CVD deaths, n(%)	3.6	1.4	2.5	4.1	7.3

### Association between whole-blood lead and all-cause and cardiovascular mortality

**Fig. 2:**
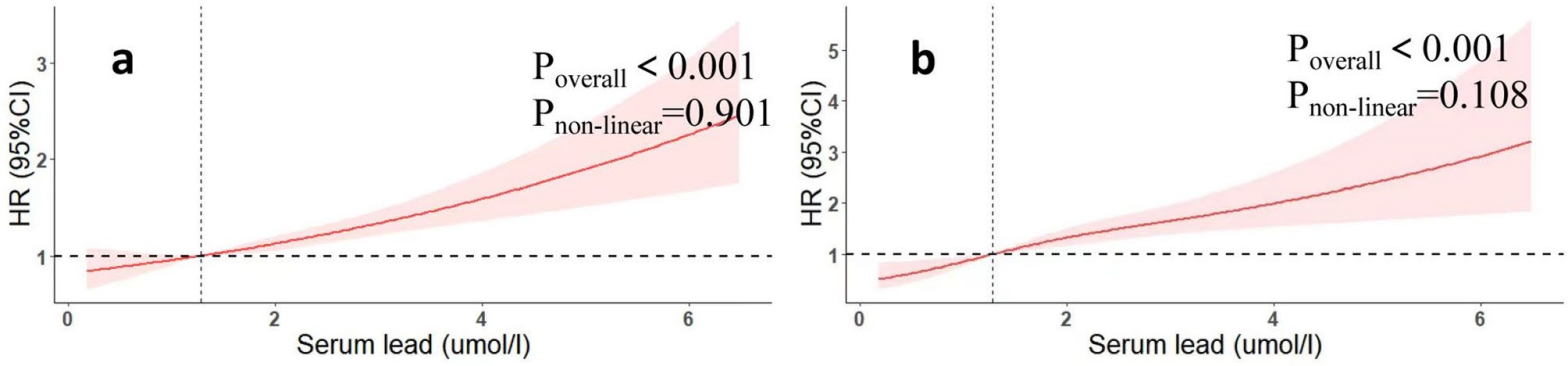
Association of whole-blood lead levels with the all-cause (**a**) and cardiovascular mortality (**b**) performed by restricted cubic spline analysis.

Table 2 shows the weighted Cox proportional risk regression results. The weighted Cox proportional risk regression showed that the risk ratio (HR) for that for all-cause mortality and cardiovascular mortality gradually increased with increasing whole-blood lead levels. Using the lowest quartile (Q1) as a reference, the HRs for all-cause mortality were 1.05 (95% CI 0.84–1.32), 1.10 (95% CI 0.89–1.36), and 1.44 (95% CI 1.16–1.79) for model 3 (Q2, Q3, and Q4), respectively. For cardiovascular mortality, they were 1.03 (95% CI 0.62–1.52), 1.30 (95% CI 0.77–2.21), and 1.97 (95% CI 1.31–2.97), respectively. Despite Q4 having the highest proportion of current smokers and the lowest proportion of higher education, the lead–mortality association persisted after detailed adjustment for smoking and education variables (Table S3).To account for the interval between smoking cessation and the event, we included years since cessation as a continuous covariate. Excluding participants who quit within the past 5 years did not alter the lead–mortality association (HR 1.47; 95% CI 1.31–1.65). All the above P trends were < 0.001. The reported baseline whole-blood lead levels in our study population were within the expected range for individuals with higher exposure levels, and all values are correctly reported in µg/dL. No unit conversion has been applied, and the elevated levels are consistent with the study population’s exposure profile. As shown in Fig. 2, restricted cubic spline regression confirmed a positive correlation between whole-blood lead concentration and risk ratio (HR) (P-overall < 0.05). Blood lead levels were positively associated with both all-cause mortality and cardiovascular.

**Table 2:** All-cause mortality and CVD-mortality hazard ratios (HRs) for participants aged 20 years and older according to malnutrition status (weighted).

Mortality outcome	Dealth/o	Weight/death (%)		Hazard ratio (95%CI)			
				Model1	Model2	Model3	
All-cause mortality	1231	11.9					
Q1	128	5.2		Ref	Ref	Ref	
Q2	250	10.3		1.86(1.47,2.34)	0.98(0.78,1.22)	1.05(0.84,1.32)	
Q3	319	13.2		2.31(1.85,2.89)	0.93(0.75,1.16)	1.10(0.89,1.36)	
Q4	534	21.3		3.87(3.09,4.85)	1.27(1.02,1.58)	1.44(1.16,1.79)	
P for trend				< 0.001	0.02	0.001	
CVD- mortality	399	3.6					
Q1	39	1.4		Ref	Ref	Ref	
Q2	72	2.5		1.76(1.15,2.68)	0.84(0.54,1.32)	1.03(0.62,1.52)	
Q3	105	4.1		2.76(1.61,4.72)	1.00(0.59,1.69)	1.30(0.77,2.21)	
Q4	183	7.3		5.11(3.25,8.04)	1.43(0.95,2.15)	1.97(1.31,2.97)	
P for trend				< 0.001	0.004	< 0.001	

CVD-mortality, cardiovascular disease mortality.

Q1(< 0.85 umol/l), Q2(0.85to 1.28 umol/l), Q3(1.28 to 2.00 umol/l). Q4(≥ 2.00 umol/l).

Model1: No adjustment.

Model2: Adjusts for basic information such as age, ethnicity and gender.

Model3: Adjust for all confounding factors.

### Survival analysis

**Fig. 3:**
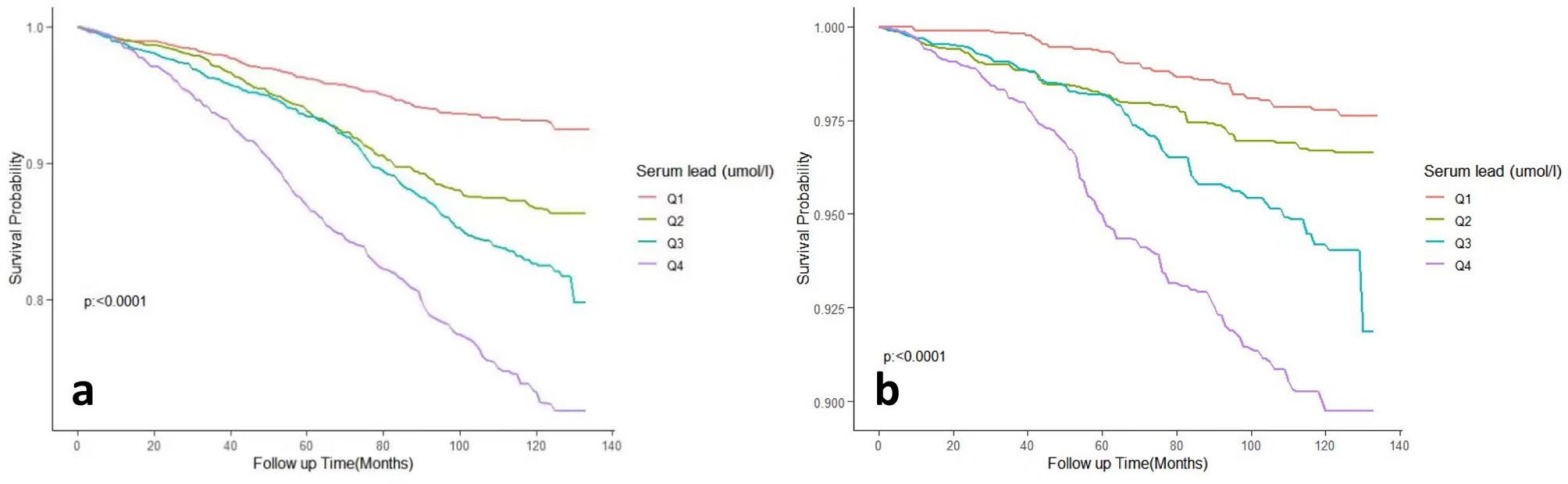
Kaplan-Meier survival curve for all-cause (**a**) and cardiovascular mortality (**b**).

As shown in Fig. 3, Kaplan Meier curves showed a significant trend of decreasing survival in the hypertensive population with increasing whole-blood lead levels (All Log-rank *P* < 0.0001).

### Subgroup analysis

The subgroup analysis of the all-cause mortality is presented in Table 3. HR was greater than 1 in all subgroups and the p-value for the interaction was greater than 0.01 in all subgroups except the education level and smoking status subgroups. This represents a strong positive link between whole-blood lead levels and all-cause mortality in different populations.

**Table 3:** Sub-group analysis.

	HR (95% CI)	*P* for interaction
Stratified by age		0.37
Age 20-40	1.86(1.30,2.66)	<0.001
Age 40-60	1.40(1.22,1.62)	<0.0001
Age 60-100	1.43(1.31,1.55)	<0.0001
Stratified by gender		0.95
Female	1.64(1.51,1.79)	<0.0001
Male	1.65(1.49,1.82)	<0.0001
Stratified by race		0.89
Mexican America	1.55(1.32,1.82)	<0.0001
Other races	1.71(1.45,2.02)	<0.0001
Non hispanic White	1.65(1.50,1.81)	<0.0001
Non hispanic Black	1.66(1.50,1.84)	<0.0001
Stratified by educational level		0.01
< High school	1.36(1.22,1.51)	<0.0001
High school	1.68(1.44,1.97)	<0.0001
> High school	1.73(1.56,1.92)	<0.0001
Stratified by BMI		0.36
< 25 kg/m^2^	1.50(1.30,1.73)	<0.0001
25–30 kg/m^2^	1.65(1.47,1.86)	<0.0001
≥ 30 kg/m^2^	1.69(1.52,1.87)	<0.0001
Stratified by smoking status		0.01
Yes	1.47(1.34,1.62)	<0.0001
No	1.81(1.62,2.03)	<0.0001
Stratified by alcohol status		0.84
Yes	1.70(1.54,1.88)	<0.0001
No	1.73(1.55,1.93)	<0.0001
Stratified by diabetes		0.53
Yes	1.62(1.45,1.82)	<0.0001
No	1.69(1.56,1.82)	<0.0001

Subgroup analyses were performed on the following covariates (age, sex, race, marital status, education level, body mass index; smoking status; alcohol consumption status; diabetes mellitus; OR, odds ratio; 95% CI, 95% confidence interval. BMI, body mass index.

## Discussion

In this retrospective NHANES-based cohort study, which included 8,364 hypertensive patients, all-cause mortality and cardiovascular mortality were 16.1% and 5.9% higher, respectively, in the highest quartile (Q4) of the whole-blood lead study population compared to the lowest quartile (Q1). Weighted Cox proportional risk regression models (adjusting for all confounders) showed an increasing trend in the risk ratio (HR) for all-cause mortality and cardiovascular mortality across the quartiles. Specifically, the HRs for all-cause mortality were 1.05 (95% CI 0.84–1.32) for Q2, 1.10 (95% CI 0.89–1.36) for Q3, and 1.44 (95% CI 1.16–1.79) for Q4. For cardiovascular mortality, the HRs were 1.03 (95% CI 0.62–1.52) for Q2, 1.30 (95% CI 0.77–2.21) for Q3, and 1.97 (95% CI 1.31–2.97) for Q4. In addition, restricted cubic spline curves confirmed a positive correlation between whole-blood lead concentration and HR (P-overall < 0.001). As whole-blood lead concentrations increased, the risk of all-cause mortality and cardiovascular death also increased. Survival curves showed a significant downward trend in survival in the hypertensive population as whole-blood lead levels increased.

In terms of educational stratification, hypertensive patients with high school education or higher had a greater risk of death due to high blood lead levels than others. People with higher education levels usually have higher social status and higher standards of living, and although a higher standard of living is usually associated with better health status, this association is variable across ethnic groups^29–32^. Therefore, we need further research to illustrate the impact of blood lead on the more educated among different ethnic groups with hypertension. In terms of smoking status stratification, hypertensive patients who did not smoke had a greater risk of death due to high blood pressure lead levels than others. Although smokers have higher absolute blood-lead levels, the slope of the exposure–response curve is steeper in non-smokers (Table 3). This pattern is consistent with previous NHANES analyses showing that environmental/occupational co-exposures to cadmium and arsenic—which are more prevalent in non-smokers living in traffic-dense or industrial areas—amplify lead-related mortality^33,34^. Smokers, despite higher lead intake from tobacco, may therefore experience a smaller incremental risk per unit increase in blood lead because their baseline risk is already elevated by multiple other constituents of tobacco smoke.Our findings suggest that blood lead levels in hypertensive populations are significantly and positively associated with mortality. Studies based on the Third National Nutrition and Health Survey (NHANES) have shown that blood lead levels remain a significant contributor to mortality in study populations with low blood lead^35^, and the latest studies have shown a positive correlation between blood lead concentrations and cardiovascular mortality^36^. A study based on the NHANES database from 2009 to 2014 showed that high exposure to lead ions was significantly associated with death from hypertension, heart disease and chronic lower respiratory disease^37^. This is consistent with our findings.

The potential possible mechanism is as follows, in a hypertensive state, the bioavailability of antioxidants is reduced, and excessive reactive oxygen species (ROS) production eventually leads to oxidative stress, and cellular and tissue damage^38^. Lead exposure causes oxidative stress in cardiovascular tissues in vivo and endothelial cells and vascular smooth muscle cells (VSMC) in vitro^3^. In addition, lead reduces nitric oxide and guanylate cyclase production in the vasculature, thereby remodeling blood vessels and inhibiting vascular relaxation^2^. Through the action of these two mechanisms, increased whole-blood lead levels exacerbate the level of oxidative stress in hypertensive patients, and oxidative stress can promote inflammation, fibrosis, and apoptosis, leading to an elevated risk of death.

Among non-smokers, blood lead predominantly reflects long-term environmental or occupational exposure, which is usually accompanied by elevated cadmium and arsenic levels. Krueger & Wade^33^ analysed NHANES 1999–2008 and demonstrated that non-smokers with chronic infections had significantly higher blood lead and cadmium concentrations than infection-free controls^33^. Wu et al.^34^ further showed that Taiwanese workers environmentally exposed to arsenic, cadmium and lead exhibited markedly elevated blood levels of all three metals^34^. Similarly, Sakellari et al.^39^ reported that residents of metropolitan Athens accumulated lead and cadmium in blood mainly through traffic-related air pollution and residential proximity to industrial emissions^40^. Collectively, these studies indicate that non-smokers are subject to mixed environmental exposures to lead, cadmium and arsenic.

In contrast, smokers derive most of their lead and cadmium burden directly from tobacco combustion. Piadé et al^41^ confirmed measurable release of cadmium and lead from tobacco to mainstream smoke—with median yields of 18.2 ng/cig (cadmium) and ＜12.8 ng/cig (lead). While not directly quantifying bloodstream transfer rates, their findings support that tobacco combustion is a key source of smoker exposure to these metals. ^41^. Consequently, smokers exhibit 4–5-fold higher blood cadmium concentrations than non-smokers, whereas arsenic co-exposure is modest. These distinct exposure patterns underscore the need for source-specific strategies in risk assessment and prevention.

Our study has several benefits. To ensure the validity of the results, we used appropriate weights and confounder adjustments during the analysis. Second, the large sample size of our study from a nationally representative population that strictly adhered to the protocol, as well as the use of a national registry to identify deaths Third, our study is the first to study the influence of whole-blood lead concentrations on all-cause mortality as well as cardiovascular mortality in hypertensive populations and is highly innovative.

However, limitations are inevitable. First, although we have adjusted for most relevant confounders, we could not exclude residual or unknown confounders. Second, we excluded numerous subjects due to missing data for some covariates, which may have led to selection bias. Third, some factors may influence lead metabolism, including dietary habits, occupational exposure, and environmental factors, which were not collected in our data.Fourth, we did not collect detailed data on the duration since smoking cessation; future longitudinal work should incorporate time-varying cessation status to clarify its impact on cardiovascular risk.

## Conclusions

Whole-blood lead concentration showed a non-linear positive correlation with all-cause mortality and cardiovascular mortality in hypertensive patients.

## Supplementary Information

Below is the link to the electronic supplementary material.

Supplementary Material 1

## Data Availability

The data that support the findings of this study are available from the first author, Yan Pu, upon reasonable request.
